# In-House Three-Dimensional Printing in Orbital Reconstructive Surgery: A Case Report and Future Perspectives

**DOI:** 10.7759/cureus.72966

**Published:** 2024-11-04

**Authors:** João Ferreira de Barros, João Oliveira, Nuno Pereira da Silva, Paulo Donato, Isabel Amado

**Affiliations:** 1 Maxillofacial Surgery, Centro Hospitalar e Universitário de Coimbra, Coimbra, PRT; 2 Radiology, Centro Hospitalar e Universitário de Coimbra, Coimbra, PRT; 3 Medicine, University of Coimbra, Coimbra, PRT

**Keywords:** 3d printing, cost-effectiveness, maxillofacial, orbital fracture, reconstructive surgery, surgical planning, trauma

## Abstract

This article discusses the application of three-dimensional (3D) printing in reconstructing the orbital floor of a middle-aged male patient who suffered an orbital fracture due to a fall. A virtual 3D model of the fractured orbit was created using open-source software, allowing for the planning of the defect reconstruction by mirroring the contralateral orbit. A metal reconstruction plate was then molded based on the 3D printed model, sterilized, and utilized during surgery, which resulted in reduced surgical time and improved adaptation. This study examines the advantages, challenges, and future potential of 3D printing in reconstructive surgery, while also relating these findings to current literature on European regulations, costs, and workflows.

## Introduction

Three-dimensional (3D) printing has revolutionized reconstructive surgery, particularly in complex cases such as orbital floor fractures. Orbital floor and medial wall fractures lead to orbital volume expansion, making surgical intervention frequent in these cases [1]. Restoring the normal anatomy of the fractured orbit without complications is challenging due to the intricate anatomical landmarks involved [1]. In complicated cases, the traditional surgical approach may be more time-consuming and can fail to achieve proper adaptation of reconstruction materials, thereby increasing the likelihood of revision surgery [2].

This article presents a successful clinical case utilizing in-house 3D printing and discusses the implications of this technology in clinical practice. We analyze the benefits and challenges of implementing this technology within a hospital setting.

## Case presentation

**Figure 1:**
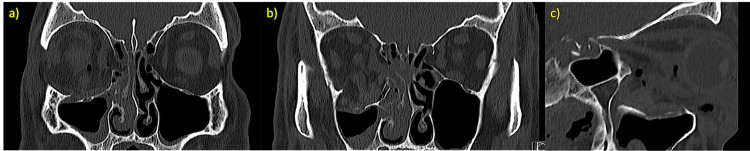
Initial CT scan demonstrating a right orbital bone fracture and herniation of orbital contents (a) Coronal view, anterior aspect. (b) Coronal view, posterior aspect. (c) Sagittal view.

In August 2022, a 55-year-old man suffered a fall from standing height, resulting in a periorbital impact. He presented to the emergency department with right orbital enophthalmos, significant periorbital edema, and complaints of binocular diplopia during horizontal gaze. A CT scan revealed a right orbital fracture with a blowout of the floor and medial wall (Figure 1). Thin slices (<1 mm) were acquired.

**Figure 2:**
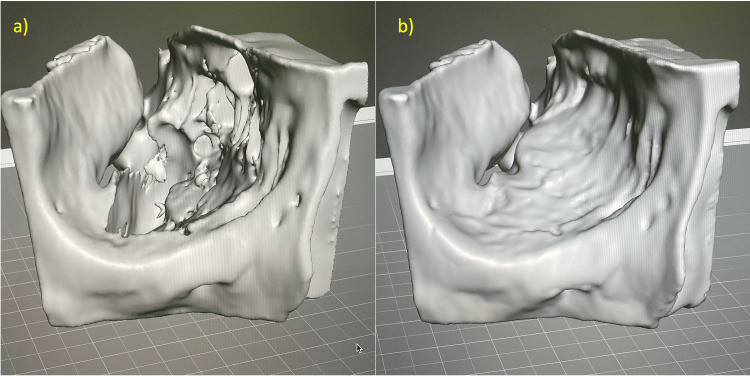
Virtual model of the affected orbit using 3D visualization software (a) Right orbital defect. (b) Superimposed image of the contralateral orbit (mirrored). 3D, three-dimensional

The patient was reevaluated five days after the trauma to assess edema resolution, and surgical intervention was indicated following confirmation of hypoglobus, limited right eye abduction, and primary binocular diplopia. Following the protocol reported by Gómez et al. [3], DICOM images from the CT scan were extracted, enabling 3D segmentation of the facial skeleton using free open-source software (3D Slicer®, version 4.11.20210226, The Slicer Community). A virtual model of the affected orbit was processed (Figure 2A), along with a superimposed image of the contralateral orbit (mirrored) for reconstruction planning (Figure 2B). Additional volume processing was performed using 3D visualization software (Autodesk Meshmixer, version 3.5.474, Autodesk Inc.).

**Figure 3:**
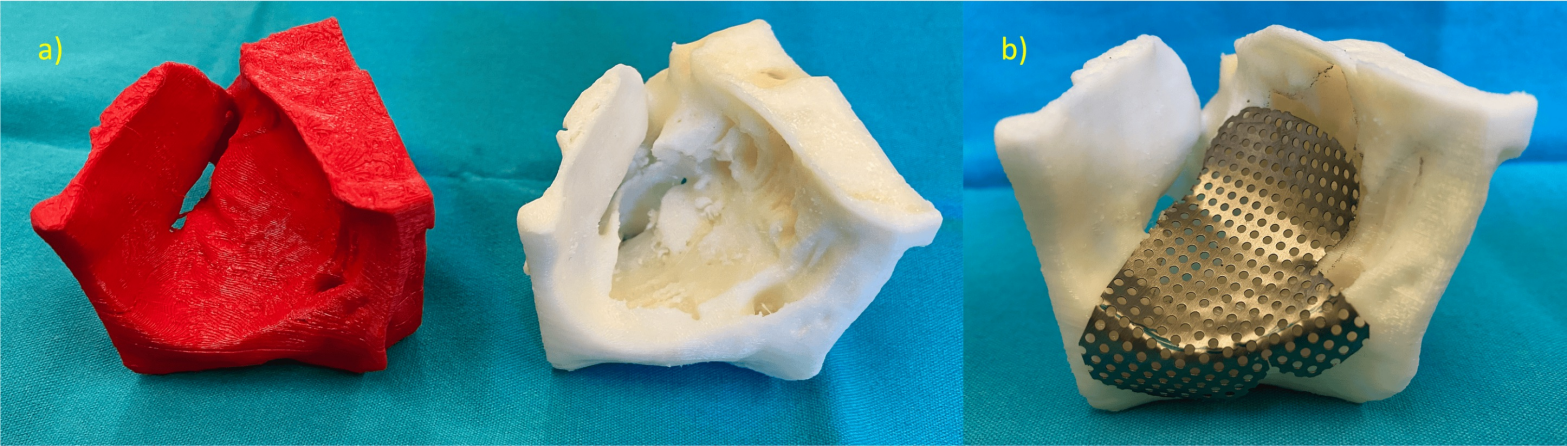
Printed material workflow (a) Printed models showing intended reconstruction (left side, red) and original traumatic defect (right side, white). (b) Molded titanium mesh for defect reconstruction (appearance before final edge trimming and polishing).

Final stereolithographic models were printed (Figure 3A) using polylactic acid (PLA-N™, Filkemp®, S.A., Portugal) filament on a 3D printer (Makerbot® Replicator™ 2, MakerBot Industries, Brooklyn, USA). A 0.2 mm thick titanium mesh (Medicon eG, Tuttlingen, Germany) was molded and adjusted to fit the defect, allowing for reconstruction in accordance with the superimposed contralateral orbit (Figure 3B).

**Figure 4:**
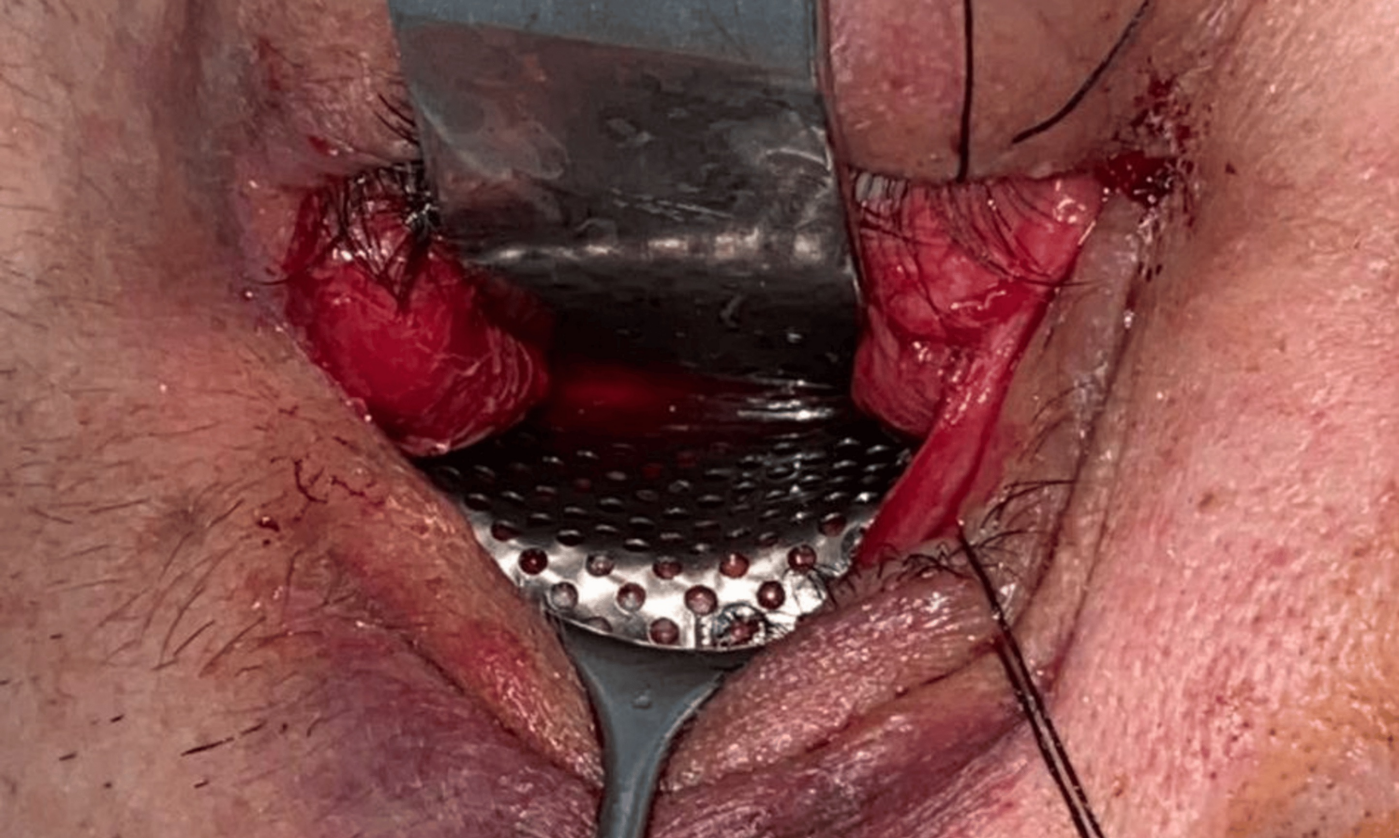
Intraoperative application of the pre-molded titanium mesh The transconjunctival approach to the right orbit, with lateral canthotomy extension, allowed for adequate surgical access.

The patient underwent surgery 13 days after the trauma. A transconjunctival approach, with lateral canthotomy extension for optimal access to the inferior orbital rim, was performed through the inferior fornix using monopolar cautery to expose the affected orbital walls. Herniated orbital contents were reduced, and the lateral bony edges of the defect were identified and isolated with periosteal elevators. The pre-formed titanium mesh was applied (Figure 4) and additionally secured with 1.6 mm osteosynthesis screws (Jeil Medical Corporation, Seoul, South Korea) along the inferior orbital rim. A forced duction test indicated no restrictions in mobility, and a sub-conjunctival sling suture was placed using 4/0 Monocryl® (Ethicon, Inc.). The lateral canthotomy extension was sutured with two simple subcuticular stitches using 4/0 Vicryl Rapid® (Ethicon, Inc.).

The intraoperative placement of the pre-molded implant was stable and adapted easily, eliminating the need for intraoperative molding. The total surgery time was 35 minutes.

**Figure 5:**
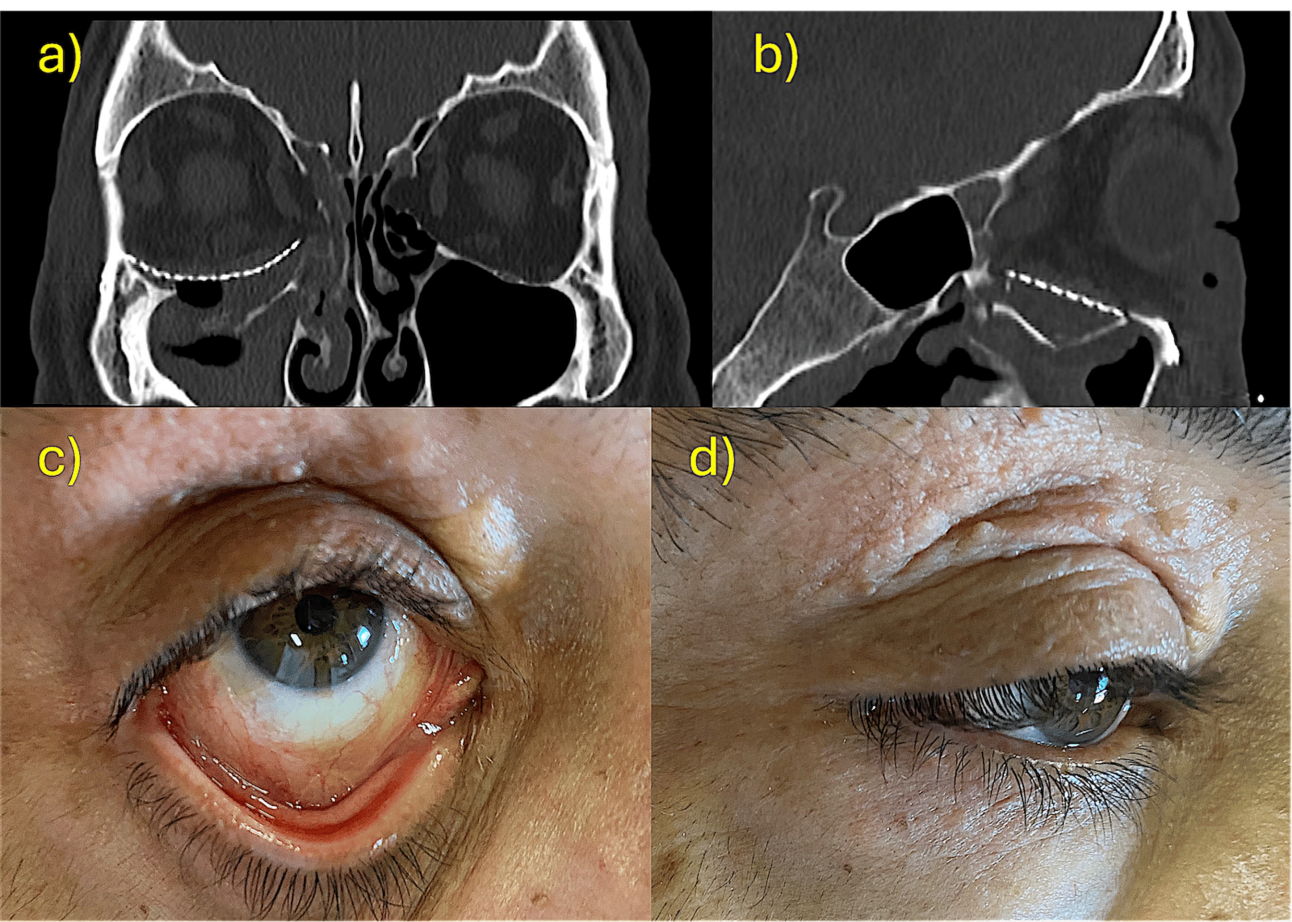
Postoperative results (a) CT scan at 24 hours postoperative (coronal view). (b) CT scan at 24 hours postoperative (sagittal view). (c) Favorable transconjunctival access scar (one month postoperative). (d) Inconspicuous lateral canthotomy extension scar (one month postoperative).

Postoperatively, the patient progressed well, with a gradual resolution of diplopia. A postoperative CT scan conducted one day after surgery demonstrated good correction of orbital volume and proper adaptation of the titanium mesh (Figure 5). The monocryl sling suture was removed after three days, while the vicryl sutures from the lateral canthotomy were left in place to allow for absorption.

## Discussion

3D printing technology, developed in the 1980s, has undergone extensive refinement and has found numerous applications in the medical field [4]. Today, advanced software and powerful computer processors enable the straightforward creation of 3D models on standard workstations [4]. Additionally, the increasing affordability and compactness of 3D printers have expanded their clinical applications across various medical specialties, offering low-cost and efficient solutions [4].

Consequently, in-house printed 3D models have become more economical and widely adopted in clinical practice, spanning specialties from maxillofacial and neurosurgery to plastic surgery, orthopedics, and cardiovascular surgery [5].

Orbital fractures often necessitate surgical correction, but these procedures are frequently delayed due to the need for periorbital edema to subside. This delay allows for more accurate palpation of the bony deformities involved and enhances the assessment of enophthalmos. Furthermore, surgical access is simplified during delayed interventions, as edema can complicate access. This waiting period presents an opportunity to prepare the necessary surgical approaches and alloplastic materials for reconstruction, which can be efficiently achieved using in-house 3D printing techniques.

Recent studies have highlighted both the controversies and advantages of 3D printing in maxillofacial and head and neck surgeries, emphasizing the importance of European regulations, costs, and timelines [6]. The literature suggests that, despite initial challenges, long-term benefits include reduced surgical times, personalized adaptations, and potential cost savings.

Our hospital’s early experiences have demonstrated that 3D printing can be highly effective in reconstructive surgery. While creating 3D models incurs upfront costs, these are significantly outweighed by long-term benefits, such as decreased surgical times and improved adaptation of reconstruction plates. In spine surgery, for instance, 3D-printed models contributed to a reduction in surgery time by 14 minutes, subsequently lowering overall healthcare costs [7]. A study on mandibular fractures revealed a notable decrease in intraoperative plating time, from 22.8 minutes to just 6.9 minutes, resulting in reduced operating room costs, which dropped from approximately $2,000 to under $700 per patient [8]. Other research indicates an average savings of 62 minutes in operating time per case in orthopedic and maxillofacial surgery, correlating with cost reductions of around $3,720 per surgery [9]. Furthermore, in head and neck reconstruction, surgery time reductions of up to 20% have been achieved with 3D printed models, enhancing plate adaptation and minimizing intraoperative trial and error, which leads to better outcomes and fewer complications [10]. This initial success has motivated the establishment of a 3D laboratory within our hospital to expand this technology to more trauma, oncology, and additional cases.

This case dates back to August 2022 and marks the first instance in our department where in-house fabricated stereolithographic models were utilized for surgical planning and shaping osteosynthesis materials. Today, stereolithographic models have become essential in our maxillofacial practice. CT DICOM files are forwarded to specialized companies that perform volume segmentation and produce patient-specific models, prostheses, or osteosynthesis materials according to the surgeons’ plans.

Although these models have been successfully employed in complex trauma and reconstruction cases, the costs and time associated with third-party planning render this option unviable for less complicated cases in daily practice. Consequently, routine cases are often managed without stereolithographic models, relying on standard-shaped osteosynthesis materials that require intraoperative adaptation to the encountered traumatic defects.

This trial-and-error approach has been the foundation of treatment since the inception of osteosynthesis, yielding satisfactory results. However, it often prolongs intraoperative time, incurs higher hospital costs, reduces the number of patients treated on the same day, and relies heavily on the surgeon’s expertise for optimal material implantation. The need for repeated insertions and removals of reconstruction material for trimming and molding increases implant fatigue, soft tissue handling (which exacerbates edema), and operative time [4].

In contrast, in-house printed 3D model-based malleable reconstruction materials (such as titanium meshes) that are pre-molded on patient-specific models are more accurate, less time-consuming, and result in less tissue damage. The total production cost of the stereolithographic models used in this case was approximately €20.

The filament used (PLA-N) possesses mechanical and thermal properties conducive to proper model sterilization. According to the Safety Data Sheet in accordance with Regulation (EC) Nº1907/2006, this filament has a melting range of 215-225 °C (419-437 °F), and thermal decomposition occurs above 300 °C (572 °F) [11]. This allows for 3D models to be adequately sterilized for use in the operating room (autoclaved at 134 °C (270 °F)), even if utilized only as support references without direct patient contact.

The printing process was completed in less than 24 hours, enabling overnight production. These figures align well with the feasibility of consistently employing this approach in daily practice.

## Conclusions

This study documents the first case conducted in our department in 2022. Since then, we have implemented this workflow in other trauma cases, as well as in bony resections related to oncologic tumor excisions and benign pathologies. Reducing operative and anesthesia times is a desirable outcome, as it enhances patient care. These in-house protocols have shown promising results in routine oro-maxillofacial surgery procedures.

In comparison to traditional methods that involve intraoperative manual bending of titanium mesh, the results of this study indicate that in-house 3D printed models offer several advantages: (a) they require minimal production time; (b) they significantly reduce intraoperative surgery time; and (c) they provide slightly better anatomical outcomes.
